# Complete Genome Sequence of a Porcine Polyomavirus from Nasal Swabs of Pigs with Respiratory Disease

**DOI:** 10.1128/genomeA.00344-18

**Published:** 2018-04-26

**Authors:** Ben M. Hause, Catherine Smith, Brian Bishop, Chelsea Stewart, Randy Simonson

**Affiliations:** a Cambridge Technologies, Worthington, Minnesota, USA; b Veterinary Medical Center, Worthington, Minnesota, USA

## Abstract

Metagenomic sequencing of pooled nasal swabs from pigs with unexplained respiratory disease identified a large number of reads mapping to a previously uncharacterized porcine polyomavirus. Sus scrofa polyomavirus 2 was most closely related to betapolyomaviruses frequently detected in mammalian respiratory samples.

## GENOME ANNOUNCEMENT

A group of preweaned pigs approximately 2 to 3 weeks of age on a farm in Minnesota presented with clinical signs of respiratory disease, mainly sneezing. Despite a history of influenza virus and porcine reproductive and respiratory syndrome virus infections, nasal swabs were collected for metagenomic sequencing to eliminate the presence of other potential pathogens.

A pool of five nasal swabs were centrifuged to remove large debris, and the supernatant was treated with a nuclease cocktail to enrich for viral nucleic acids. Barcoded random primers were used for reverse transcription and second-strand synthesis followed by amplification using a barcode primer. Sequencing libraries were prepared using an Illumina Nextera XT kit and sequenced on an Illumina MiniSeq system using paired 150-bp reads. The resulting 3.1 million reads were mapped to the host Sus scrofa genome, with the unmapped 2.5 million reads next assembled *de novo* into 550 contigs at least 1,000 bp in length, which were analyzed by BLASTn.

A 4,978-bp contig consisting of over 20% of the reads showed sequence similarity to a recently described alpaca polyomavirus (1) (GenBank accession number KU879245). Additional viruses associated with porcine respiratory disease identified in the pooled nasal swabs included porcine cytomegalovirus (2), porcine astrovirus 4 (3), and porcine parainfluenza virus 1 (4). The presence of multiple respiratory viruses and lack of additional diagnostic testing preclude assignment of an etiologic role to any of these viruses for the observed respiratory disease.

BLASTp analysis of the putative large T antigen found 66% identity to alpaca polyomavirus, indicating that this virus represents a novel species, Sus scrofa polyomavirus 2 (5). Likewise, predicted proteins for the small T antigen and VP1 and VP2 capsid proteins were most similar to alpaca polyomavirus (49 to 68% identity). In addition to sequence similarity, phylogenetic analysis supports Sus scrofa polyomavirus 2 as a new species in the genus Betapolyomavirus.

Betapolyomavirus includes 26 recognized species that infect mammals (5). Interestingly, the highest observed sequence similarity was to alpaca polyomavirus and human polyomavirus 3 and 4 (KI and WU polyomaviruses, respectively), all of which have been associated with respiratory disease (1, 6–9). Further research is needed to determine the clinical significance of Sus scrofa polyomavirus 2 infection of pigs.

### Accession number(s).

The complete genome sequence of Sus scrofa polyomavirus 2 (strain 180230) was deposited in GenBank under accession number MG976810.
